# Advances in the application of artificial intelligence in ophthalmic education and clinical training

**DOI:** 10.3389/fmed.2025.1753411

**Published:** 2026-01-13

**Authors:** Mingsi Chi, Ying Cui, Lei Xi

**Affiliations:** Department of Ophthalmology, Guangdong Provincial People’s Hospital, Guangdong Academy of Medical Sciences, Southern Medical University, Guangzhou, Guangdong, China

**Keywords:** artificial intelligence, clinical reasoning, computer vision, large language models, medical education, ophthalmology, surgical training

## Abstract

Ophthalmic education faces increasing demands due to rising disease burden, prolonged training pathways, and unequal access to educational resources. Artificial intelligence (AI) is increasingly used to support ophthalmic training across multiple educational stages. This review summarizes recent evidence on AI applications in ophthalmic education, focusing on theoretical knowledge assessment and content generation, the objective evaluation of microsurgical skills, AI-assisted development of clinical diagnostic reasoning, and patient education. Large language models enable scalable knowledge assessment and rapid generation of structured educational materials, while computer vision and sensor-based technologies provide objective, quantitative feedback for microsurgical training. AI-assisted diagnostic and simulation systems support clinical reasoning through visual explanations and diverse virtual cases, and AI-driven tools improve the accessibility and readability of patient’s education materials. However, ethical and practical challenges—including model hallucination, data bias, privacy risks, and implementation barriers—limit widespread adoption. Addressing these issues through robust governance and effective human–AI collaboration is essential for safe, equitable, and high-quality ophthalmic education.

## 1 Introduction

Ophthalmology, a specialty that relies heavily on image interpretation, refined microsurgical techniques, and clinical reasoning, is positioned at the forefront of technological innovation driven by artificial intelligence (AI). With global population aging, the prevalence of vision-threatening diseases such as diabetic retinopathy, age-related macular degeneration, and glaucoma continues to increase, placing growing demands on ophthalmic training systems. Meanwhile, prolonged training pathways and uneven distribution of educational resources remain major challenges, particularly in low- and middle-income regions. Traditional apprenticeship-based training models, which depend heavily on case availability and subjective expert assessment, are increasingly insufficient to meet the need for standardized, efficient, and scalable ophthalmic education. Recent advances in AI, including deep learning, computer vision, and generative models, have demonstrated strong capabilities in medical image analysis, procedural assessment, and knowledge processing. While these technologies were initially developed for clinical diagnosis and decision support, they are currently being actively adapted for educational purposes in ophthalmology (1–3).

AI systems can support theoretical learning, enable the objective evaluation of microsurgical skills, assist in the development of clinical diagnostic reasoning, and enhance patient education, thereby addressing key limitations of conventional training paradigms. Generative AI systems, particularly large language models (LLMs), show potential in knowledge assessment, educational content generation, and simulated clinical dialogue. In parallel, computer vision and sensor-based technologies enable the quantitative analysis of surgical performance, transforming skill assessment from subjective judgment to objective measurement (4, 5). In parallel, computer vision and sensor-based technologies enable the quantitative analysis of surgical performance, transforming skill assessment from subjective judgment to objective measurement (6, 7). Additionally, AI-assisted diagnostic and simulation platforms further facilitate clinical reasoning by providing visual explanations, interactive feedback, and exposure to diverse virtual cases (8–11).

**Figure 1:**
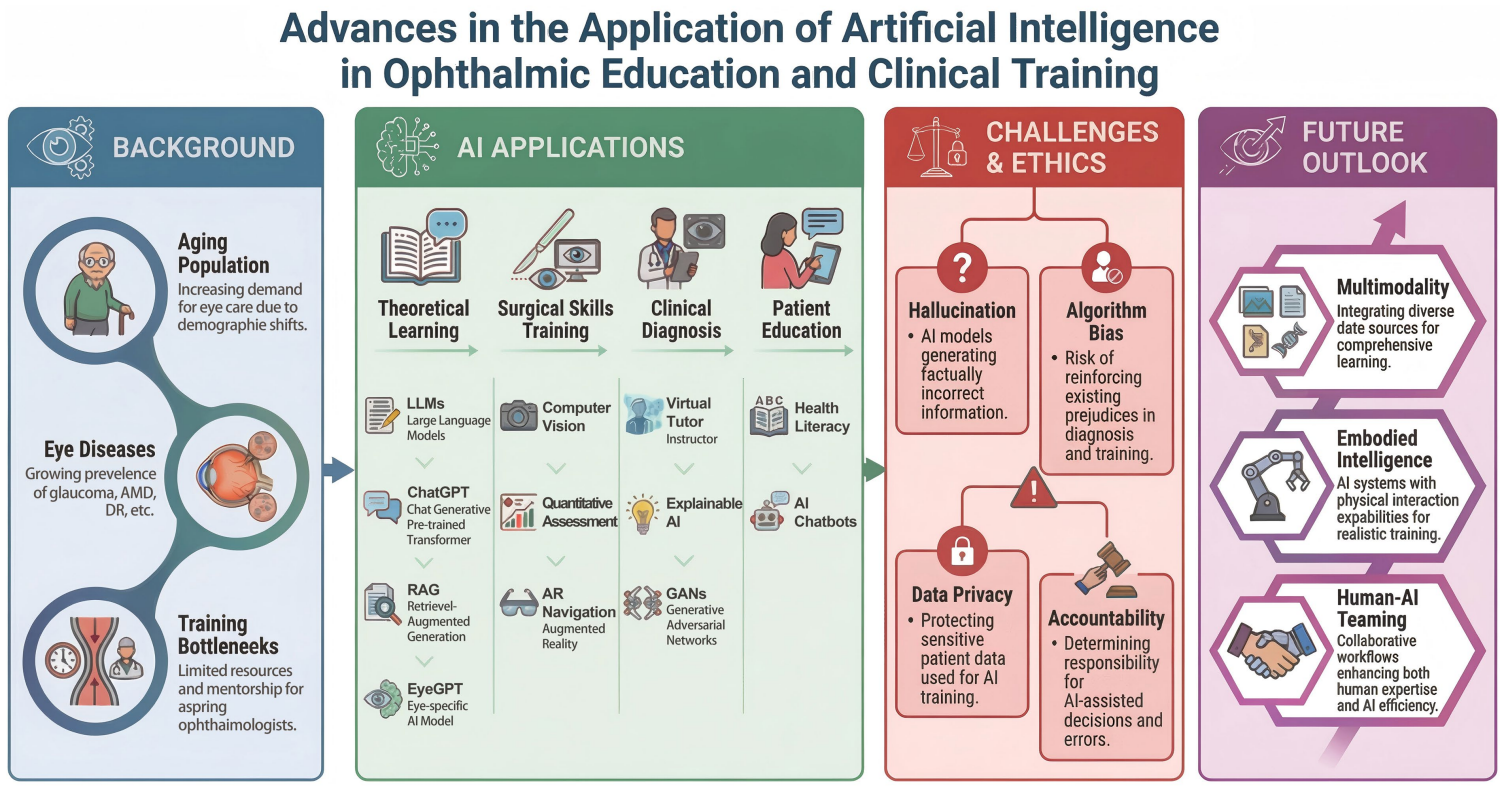
Advances in the application of artificial intelligence in ophthalmic education and clinical training.

Despite these promising prospects, the deep integration of AI into ophthalmic education faces multiple challenges, including the risk of algorithmic “hallucinations,” concerns over data privacy, questions regarding ethical accountability, and issues related to model interpretability (12). In this review, “generative AI” refers broadly to AI systems capable of producing novel content across text, image, or multimodal domains, whereas LLMs represent a specific subclass of generative AI designed primarily for natural language understanding and generation. This review systematically synthesizes recent evidence on AI-assisted ophthalmic education across defined educational domains, analyzes associated ethical and implementation challenges, and discusses future directions aimed at supporting safe, equitable, and high-quality ophthalmic training (Figure 1).

## 2 Literature search strategy

A systematic literature search was conducted across multiple electronic databases, including PubMed, Scopus, and Web of Science, including publications from January 2024 to November 2025. The search strategy combined controlled vocabulary (MeSH terms) and free-text keywords as follows:

(“artificial intelligence” OR “large language models” OR “deep learning” OR “computer vision”) AND (“ophthalmology” OR “ophthalmic”) AND (“education” OR “training” OR “surgical skill” OR “clinical reasoning”).

The inclusion criteria were (1) original research articles, reviews, or clinical trials focusing on applications of AI in ophthalmic education and (2) studies published in English.

The exclusion criteria were (1) studies in which AI was applied solely for clinical diagnosis or decision-making without an educational component and (2) abstracts, editorials, commentaries, and other non-peer-reviewed publications.

Two authors independently screened titles and abstracts, followed by a full-text review to determine final eligibility. Disagreements were resolved through discussion with a third author. The methodological quality of included studies was assessed using established checklists appropriate to their respective study designs.

## 3 Applications of generative artificial intelligence in theoretical knowledge assessment and educational content development in ophthalmology

Generative artificial intelligence has rapidly expanded its role in medical education, shifting from basic information retrieval toward structured knowledge assessment and educational content development. Within ophthalmology, this transition is particularly relevant due to the discipline’s strong reliance on guideline-based knowledge, image interpretation, and standardized theoretical training. Among generative AI approaches, large language models (LLMs) have attracted significant attention for their capacity to process complex ophthalmic text, to simulate clinical reasoning, and to generate structured educational materials. For example, the application of the AI-based pathologic myopia identification system showed advantages in promoting the efficiency of the ophthalmology residency training program (13).

Several studies have evaluated the performance of general-purpose LLMs in ophthalmology-specific knowledge assessments, primarily using board-style or guideline-based examination questions. Overall, these models demonstrate strong competence in text-based theoretical tasks, with several studies reporting accuracy levels comparable to, or exceeding, those of ophthalmology trainees across multiple subspecialties and cognitive complexity levels. Study shows that OpenAI’s o1 model achieved an accuracy of 84.6% on ophthalmology board-style questions—significantly outperforming GPT-4o (66.2%) and Gemini 1.5 Flash (60.2%)—and excelled across all ophthalmic subspecialties and levels of cognitive complexity (14). Another comparative study of 500 questions further supported these findings, with the o1 Pro model attaining an accuracy of 83.4%, surpassing DeepSeek R1 and Grok 3, and demonstrating robust reasoning capabilities in addressing complex ophthalmic problems (15). In the field of cataract and refractive surgery, ChatGPT-4o achieved 84–86% accuracy on *BCSC* self-assessment items, making it the only model statistically superior to ophthalmology residents (16). Additional research has shown that domain-specific models, such as EYE-Llama, outperform general-purpose Llama 2 and ChatGPT on ophthalmology-focused questions, underscoring the value of specialty-specific pretraining and fine-tuning in improving educational accuracy (17). Despite their strong performance on text-only questions, substantial limitations emerge when LLMs are applied to image-dependent theoretical assessments. Studies consistently report a decline in accuracy when visual interpretation is required, reflecting current constraints in multimodal integration and visual reasoning (18–20). These findings indicate that, while LLMs are suitable for supporting guideline-based knowledge evaluation, they remain insufficient as standalone tools for the image-intensive theoretical assessment in ophthalmology.

Beyond functioning as assessment tools, generative AI also plays an expanding role in educational content development. The creation of high-quality clinical cases and multiple-choice questions (MCQs) is traditionally resource-intensive; in contrast, LLM-based systems can rapidly generate guideline-aligned practice materials. Although early evaluations demonstrate low textual overlap between AI-generated and existing question banks, expert reviewers generally rate AI-generated MCQs as comparable in clarity, relevance, and educational appropriateness, supporting their use as supplementary resources rather than replacements for expert-developed materials (21). Moreover, clinical case scenarios containing MCQs generated by ChatGPT 4.0 have been successfully incorporated into undergraduate medical revision courses, with 95% of students reporting that the materials enriched their learning experience. However, simultaneously identifying variability in content quality and occasional factual inaccuracies underscores the continued need for expert review and curation (4).

To overcome the limitations of general-purpose LLMs in ophthalmic education, several domain-specific systems have been developed. For example, the OphGLM framework integrates an image encoder, text encoder, and LLM backbone, supported by a Chinese ophthalmology-specific fine-tuning dataset. This system enables low-cost incorporation of visual understanding and ophthalmic knowledge, demonstrating strong performance in visual reasoning and interactive tasks (22). In contrast, EyeGPT emphasizes text-based ophthalmic knowledge through domain-specific fine-tuning, retrieval-augmented generation, and role-based prompt engineering, aiming to reduce hallucinations and improve explanatory consistency in clinical question answering (23). Additionally, an LLM-based digital patient system (LLMDP) has been developed to support history-taking training. By simulating authentic patient–physician conversations, LLMDP significantly improves students’ performance in history-taking assessments and enhances empathy, thereby providing a scalable, low-risk supplement to limited clinical case exposure (24).

Collectively, current evidence suggests that generative AI can support ophthalmic theoretical education by assisting knowledge assessment, expanding educational content repositories, and facilitating simulated learning interactions. However, due to persistent risks of factual error and variability in output quality, these tools should be integrated as adjuncts to, rather than replacements for, expert-led education, with human-in-the-loop oversight remaining essential for educational reliability and safety (4, 21) (Table 1).

**Table 1:** Summary of representative studies on the theoretical knowledge and content generation of AI in ophthalmology education.

AI Technology	Evaluation metrics	Key findings	References
OpenAI o1	Accuracy and performance stratified by ophthalmic subspecialty and cognitive complexity level	OpenAI o1 demonstrated superior performance over GPT-4o, Gemini, and human test takers in answering ophthalmology board-style questions, which were drawn from two question banks and stratified across three levels of complexity.	(14)
		OpenAI o1 Pro excelled in questions spanning eight of nine ophthalmologic subfields, tasks requiring second- and third-order cognitive complexity, and image-based assessments.	(15)
GPT-4	Answering cataract and refractive surgery questions	Among the tested models, ChatGPT-4o achieved the highest accuracy with a simple prompt (84%; 95% CI: 77–91%), followed by Gemini Advanced (82%), Copilot (78%), ChatGPT-4 (77%), and Gemini (62%), with the differences being statistically significant (*p* < 0.05).	(16)
EYE-Llama	Accuracy and reliability question–answering systems	EYE-Llama achieved superior scores on evaluation metrics, including the BERT (Bidirectional Encoder Representations from Transformers) score, BART (Bidirectional and Auto-Regressive Transformer) score, and BLEU (Bilingual Evaluation Understudy).	(17)
OphGLM	Ophthalmic visual diagnostic interaction	Leveraging the FundusTuning-CN dataset, OphGLM surpasses open-source medical LLMs in both specialized ophthalmic knowledge and interactive functions.	(22)
EyeGPT	Enhance ophthalmic knowledge	The optimized fine-tuned model significantly outperformed the original Llama2 by delivering more informed advice (mean 9.30, SD 4.42 vs. mean 13.79, SD 5.70; *p* < 0.001) and showing a marked reduction in hallucinations (non-hallucinatory rate: 80.8% vs. 44.2%; *p* < 0.001).	(23)
LLMDP	Practice medical history-taking skills	A single-center randomized controlled trial demonstrated that training with LLMDP significantly improved students’ medical history-taking scores by 10.50 points (95% CI: 4.66–16.33, *p* < 0.001) and enhanced their empathy relative to traditional methods.	(24)

## 4 Objective assessment and training of ophthalmic microsurgical skills using computer vision and sensor-based technologies

Ophthalmic surgery is characterized by its precision, microscopic operating field, and minimal tolerance for error. Traditional “observe–practice–teach” models are not only limited in efficiency but also lack standardized, objective metrics for evaluating surgical performance. With advances in computer vision and sensor technologies, AI-driven surgical video analysis and motion-capture systems are transforming microsurgical training by providing quantitative, reproducible assessment tools.

Surgical video analysis currently represents the most widely implemented technological pathway. Deep learning-based systems can automatically perform surgical phase recognition and instrument segmentation, enabling the objective evaluation of procedural efficiency and adherence to standardized workflows. In cataract phacoemulsification, AI platforms have demonstrated the ability to identify and analyze surgical phases in real time, establishing efficiency benchmarks for both consultants and trainees and even offering intraoperative phase detection feedback to help reduce operative time and optimize team coordination (6). These approaches are particularly valuable in addressing global disparities in surgical training. Manual small-incision cataract surgery (MSICS), widely practiced in resource-limited regions due to its cost-effectiveness and minimal equipment requirements, faces substantial training challenges stemming from limited expert supervision and highly subjective assessment methods. To mitigate these barriers, an Multi-Stage Temporal Convolutional Network architecture has been developed and evaluated on the SICS-105 dataset, achieving high accuracy in MSICS phase recognition. Although phase identification in MSICS remains more challenging than in phacoemulsification due to greater procedural complexity, this study establishes a foundation for standardized, scalable training in underserved settings (25). By deconstructing the complex surgical procedure into quantifiable metrics, the AI system can supplement expert instruction, offer trainees consistent and objective feedback, and thereby accelerate their learning curve while ensuring procedural standardization.

Beyond workflow-level analysis, AI systems have also been developed for the fine-grained evaluation of specific surgical maneuvers. The CatSkill system uses deep learning to segment anatomical structures (such as the limbus and Purkinje images) and instruments in surgical videos, generating objective metrics—collectively termed CatSkill Assessment Metrics (CSAMs)—that quantify parameters such as globe centration and microscope focus quality. Studies show that attending surgeons score higher than residents across all CSAMs, and machine-learning models trained solely on these metrics can reliably distinguish skill levels (AUC 0.865), supporting their use for automated resident assessment (26). Similarly, the PhacoTrainer system analyzes trajectory length, velocity, and instrument movement regions, with AI-generated metrics showing strong correlations with expert Objective Structured Assessment of Cataract Surgical Skill (OSACSS) scores. This approach effectively differentiates novices from experts and identifies common deficiencies in junior trainees, including difficulty maintaining globe and probe centration. It also effectively differentiates novices from experts and identifies common deficiencies in junior trainees, including difficulty maintaining globe and probe centration (27).

In more complex corneal transplant procedures, real-time navigation and augmented reality (AR) technologies are emerging as advanced training tools. A joint-learning iterative module has been developed to track and restore corneal images under severe occlusion, enabling the construction of an AR-guided system that assists surgeons in planning suture placement more precisely during deep anterior lamellar keratoplasty (DALK) (28). In addition, for intraocular lens (IOL) positioning during phacoemulsification, neural network–based microscope systems have been designed to provide end-to-end real-time navigation, accurately locating the ocular center and tracking rotational movements to support high-precision surgical tasks (29).

For example, multimodal tracking data—including gaze patterns and hand gestures—can distinguish collaboration styles in virtual reality (VR) surgical tasks, offering implications for training teamwork and communication skills (30). Wearable motion-capture devices have also been used to detect eye-rubbing behaviors through machine-learning algorithms, suggesting potential applications in identifying and correcting unwanted micro-movements during surgical simulation training (31, 32). Furthermore, advances in visual servoing enable robot-assisted systems to automatically adjust instrument positioning based on real-time image feedback. Although currently applied primarily in surgical assistance, these technologies provide technical foundations for future automated surgical training platforms (33). Beyond video analysis, sensor technologies and eye-tracking systems are being integrated into surgical skill assessment, particularly within simulated environments. These modalities provide insights into non-verbal behavior, motor coordination, and cognitive load. For example, in virtual reality (VR) collaborative tasks, multimodal tracking data—including gaze behavior and hand gestures—can distinguish between various collaboration patterns, providing valuable implications for training teamwork and communication in surgical settings (30). Wearable motion-capture devices have also been used to detect eye-rubbing behaviors through machine-learning algorithms, suggesting potential applications in identifying and correcting unwanted micro-movements during surgical simulation training (31, 32). Furthermore, advances in visual servoing enable robot-assisted systems to automatically adjust instrument positioning based on real-time image feedback. Although currently applied primarily in surgical assistance, these technologies provide technical foundations for future automated surgical training platforms (33).

Collectively, AI-driven objective assessment systems are increasingly augmenting traditional subjective evaluations in ophthalmic microsurgery. By converting surgical workflows and movements into quantifiable data streams, these technologies enable immediate, objective feedback for trainees and allow educators to identify specific performance deficits. Such data-driven approaches support precise and personalized skill development and represent a critical step toward a standardized, scalable surgical training program in ophthalmology (Table 2).

**Table 2:** Summary of representative studies on the objective assessment and training of ophthalmic microsurgical skills of AI in ophthalmology education.

AI technology	Evaluation metrics	Key findings	References
CatSkill	Surgeon proficiency in maintaining eye neutrality, eye centration, and adequate focus	The CatSkill system, utilizing a Feature Pyramid Network (FPN) with a VGG16 backbone, accurately segmented the palpebral fissure, limbus, and first Purkinje image, achieving a Dice coefficient of 94.03%. Subsequently, a random forest model trained on these surgical assessment metrics achieved an AUC of 0.865 in differentiating surgeon skill levels between attending and resident physicians.	(25)
PhacoTrainer	Cataract surgical skill performance in phacoemulsification probe decentration, eye decentration, and zoom level change	A significant negative correlation (*r* values from −0.77 to −0.49) was identified between AI-generated metrics and the Objective Structured Assessment of Cataract Surgical Skill (OSACSS) sub-items that evaluate eye centration and various surgical steps.	(27)
Augmented reality	Clear and unoccluded view of the corneal regions	The Iter-S model demonstrated high performance, achieving a mean endpoint error of 1.69, a peak signal-to-noise ratio (PSNR) of 36.86, and a structural similarity index measure (SSIM) of 0.976, all with a rapid inpainting inference time of 16.26 ms.	(28)
Neural network–based microscope systems	Surgical planning and navigation	The EyeNavNet, an intraoperative end-to-end navigation network, was developed for real-time eye center localization and rotation tracking. When tested on a large real-world clinical dataset, the algorithm achieved a low position error of 0.121 ± 0.044 mm and a rotation error of 1.07 ± 0.50°.	(29)
Virtual reality	Communication and collaboration	Communication patterns in virtual reality collaboration varied by task constraints: gestures were more prevalent in shared-visual tasks, while speech became longer and more structured when turn-taking was enforced. Notably, joint attention improved when verbal descriptions were used instead of a shared visual reference.	(30)
Wearable devices	Eye rubbing	The model achieved 94% accuracy, enabling the developed application to successfully recognize, count, and display the number of eye-rubbing events, providing a tool to significantly improve management for patients with keratoconus and those undergoing refractive surgery.	(31)
		The algorithm’s accuracy for detecting eye-rubbing scales with the duration of user-specific fine-tuning, exceeding 80% after 20 min (minimal) and reaching up to 97% after 3 h (moderate), leading to further studies and patient education in keratoconus management.	(32)

## 5 AI-assisted development of clinical diagnostic thinking and simulation-based teaching systems

Cultivating clinical diagnostic reasoning is a core objective of medical education, aiming to train physicians to extract key features from complex clinical information and form rational differential diagnoses. In ophthalmology, deep learning-based computer-aided diagnosis (CAD) systems and simulation platforms have increasingly been adopted to support this process. Importantly, contemporary educational AI systems extend beyond providing diagnostic outputs; they also offer visual explanations, interactive feedback, and guided learning pathways that help trainees internalize expert reasoning patterns.

AI-assisted diagnostic systems, functioning as “virtual mentors,” enhance diagnostic accuracy while accelerating learning curves through human–AI collaboration. A multicenter study demonstrated that iterative training using AI-assisted labeling significantly improved ophthalmologists’ diagnostic accuracy for nine common retinal diseases, with performance gains increasing across successive annotation rounds (8). In anterior segment disease diagnosis, the CorneAI model increased residents’ accuracy from 75.6 to 86.2%, demonstrating notable teaching benefits even when using smartphone-captured images (9). Similarly, for eyelid tumor identification, deep learning-based mobile applications provide high classification accuracy along with intuitive interfaces and treatment recommendations, assisting general practitioners and residents in differentiating benign lesions from malignant ones (34). Beyond these examples, AI-assisted diagnostic tools have also been applied to ocular surface diseases and diabetic retinopathy, contributing to improved access to eye care and partially mitigating regional shortages of ophthalmic expertise (35, 36).

In addition to supporting real-world diagnosis, AI enables simulation-based education by expanding access to rare and complex cases. Generative adversarial networks (GANs) can synthesize high-quality ophthalmic images, addressing the scarcity of representative training data. For instance, AI-generated images of infectious keratitis have been shown to improve medical students’ diagnostic accuracy as effectively as real cases, providing a virtually unlimited and diverse case library for standardized clinical training (37). Similarly, for retinopathy of prematurity (ROP), a severe blinding condition, AI-integrated telemedicine simulation platforms help address the global shortage of trained personnel by recreating real screening scenarios and enhancing trainees’ ability to detect subtle lesions (38).

The “explainability” of AI systems plays a crucial role in education. Through heatmaps or saliency maps, AI can highlight the rationale behind its decisions, directing learners’ attention to critical lesion areas. In glaucoma assessment, deep learning models trained on expert eye-tracking data can predict diagnostically meaningful fixation zones on optical coherence tomography (OCT) images, enabling novice clinicians to more efficiently identify key structural changes (39). Similar expert-guided visual search strategies have demonstrated significant educational benefits in radiology, improving diagnostic accuracy among trainees (40, 41). However, learners’ responses to explainable AI vary by level of experience. While explainability enhances trust and confidence among experienced clinicians, inexperienced learners may over-rely on incorrect AI outputs, underscoring the need for carefully designed feedback mechanisms that promote critical appraisal rather than passive acceptance (42).

Furthermore, the emergence of interactive reasoning agents (Reasoning Agents) elevates AI support from simple image recognition to logical reasoning. ReasonAgent integrates multimodal visual analysis, knowledge retrieval, and stepwise diagnostic reasoning modules, demonstrating superior treatment-planning performance for complex and rare ophthalmic conditions compared with general-purpose LLMs and some resident physicians. By explicitly modeling intermediate reasoning steps, such systems represent a promising paradigm for teaching clinical thinking rather than merely delivering diagnostic conclusions (43).

Collectively, these developments indicate that AI is evolving from a passive diagnostic aid into an interactive educational partner capable of guiding reasoning, simulating expert decision-making, and providing targeted feedback. When appropriately integrated into training curricula, AI-assisted diagnostic and simulation systems can support the development of accurate, reflective, and adaptable clinical reasoning skills in ophthalmology (Table 3).

**Table 3:** Summary of representative studies on the clinical diagnostic thinking and simulation-based teaching systems of AI in ophthalmology education.

AI technology	Evaluation metrics	Key findings	References
Artificial intelligence reading label system	Diagnostic accuracy of retinal diseases	In a human–AI collaborative training model, the average diagnostic accuracy for nine retinal diseases and normal fundus improved significantly over five rounds (*p* = 0.013). Furthermore, accuracy was found to be closely correlated with the participants’ duration of ophthalmology study (*p* = 0.007).	(8)
CorneAI model	Ophthalmologists’ diagnostic accuracy	The CorneAI model demonstrated significant teaching benefits by increasing the diagnostic accuracy of specialists from 82.8 to 90.0% and residents from 75.6 to 86.2% (*p* < 0.001). This resulted in a rise in the overall accuracy for ophthalmologists from 79.2 to 88.8% (*p* < 0.001).	(9)
Intelligent Eyelid Tumor Screening	Self-diagnosing eyelid tumors	A smartphone-based application, developed from the top-performing model, achieved 92.1% accuracy for triple classification (benign/malignant eyelid tumors or normal eye) on an external validation dataset. This level of accuracy was generally superior to that of general physicians, resident doctors, and even ophthalmology specialists.	(34)
Generative adversarial networks	Diagnostic accuracy of keratitis	The students using the real cases for teaching had the highest mean accuracy improvement (28.43%).	(37)
ReasonAgent	Ophthalmic decision-making	ReasonAgent excelled in treatment planning, significantly outperforming GPT-4o (*β* = 0.49, *p* = 0.01) and ophthalmology residents (*β* = 1.71, *p* < 0.001), especially in rare disease cases (all *p* < 0.05). In contrast, its diagnostic accuracy was on par with that of residents (*β* = −0.07, *p* = 0.65).	(43)

## 6 The role of artificial intelligence in ophthalmic patient education and health literacy enhancement

Patient education is a crucial component of ophthalmic healthcare, as adequate health literacy is closely linked to improved adherence and treatment outcomes. However, traditional physician–patient communication is often constrained by limited time, and existing online health information is frequently overly complex. Recent advances in artificial intelligence (AI), particularly large language models (LLMs) and conversational chatbots, offer new opportunities to enhance the accessibility and effectiveness of patient education.

AI tools such as ChatGPT have demonstrated remarkable capability in generating easily understandable patient education materials (PEMs). Studies have shown that ChatGPT-4 and its optimized versions can rewrite complex ophthalmology literature or existing PEMs to a readability level suitable for the general public, significantly improving comprehension while maintaining content accuracy and high quality (11, 44). In dry eye disease education, materials generated by ChatGPT-4 were found to be highly readable and almost error-free, making them an effective supplement to conventional educational resources (45). For myopia, a global visual health issue, the ChatMyopia AI agent integrates image classification and retrieval-augmented knowledge bases; randomized controlled trials demonstrated that it outperformed traditional pamphlets in enhancing patient satisfaction, disease awareness, and empathy (10).

Beyond static educational content, AI systems also exhibit strong potential in disease-specific patient question-and-answer interactions. In thyroid eye disease (TED) education, LLM-generated brochures and frequently asked questions have been shown to surpass standard internet search results in terms of quality, comprehensibility, and empathetic tone (46). In conditions such as glaucoma, uveitis, and cataracts, ChatGPT-generated responses are generally evaluated by experts such as accurate, coherent, and safe, although there remains room for improvement in comprehensiveness and individualized advice (47–50). Notably, in patient triage scenarios, ChatGPT-4 showed high concordance with ophthalmologists in assessing urgency and recommending appropriate clinical pathways, sometimes even favoring safer recommendations, highlighting its potential for triage support and preliminary consultation (51, 52).

However, AI applications in patient education face challenges related to language and cultural context. A study on pediatric myopia counseling in Chinese found that only 35% of ChatGPT responses fully adhered to standards, indicating that AI performance may be substantially reduced in non-English or culturally specific contexts, necessitating targeted optimization and validation (53). Furthermore, while AI can provide informational support, it cannot fully replace professional clinical judgment in complex treatment decisions or individualized care recommendations (54, 55).

Overall, AI is emerging as a powerful tool for enhancing ophthalmic health literacy. It offers round-the-clock, personalized, and comprehensible health information, lowering barriers to specialized knowledge. Nevertheless, ongoing monitoring and optimization are essential to ensure content accuracy and applicability.

## 7 Ethical challenges and implementation barriers in AI-assisted ophthalmology education

Despite the tremendous potential of AI in ophthalmic education and clinical applications, its widespread adoption faces significant ethical challenges and implementation barriers. These concerns extend beyond technical performance and encompass issues of reliability, fairness, privacy protection, accountability, and alignment with the core values of medical education.

One of the most prominent risks in educational applications is AI “hallucination.” Generative models may produce confident but factually incorrect information, fabricated references, or internally inconsistent explanations when generating ophthalmology-specific content or simulated clinical cases. Studies have reported discrepancies between AI-generated imaging descriptions and diagnostic reasoning, which—if incorporated into teaching materials without adequate oversight—may mislead trainees and distort clinical reasoning development (4). Moreover, AI models may misinterpret or inaccurately explain data; if such uncertainties are not adequately communicated, they could introduce risks in clinical decision-making (12). To mitigate this risk, establishing a multi-layered quality control framework is crucial. This could involve, first, automated cross-validation of AI-generated content against established clinical guideline knowledge bases, and second, mandatory manual review by senior ophthalmologists before deployment in curricula. Furthermore, embedding “confidence scores” or “uncertainty labels” in AI outputs can effectively prompt learners to maintain a critical perspective rather than passively accepting the information. These measures help reinforce critical appraisal skills rather than passive reliance on AI-generated information.

Data bias and algorithmic fairness represent another major ethical concern. Current ophthalmic AI models are often trained on datasets lacking demographic diversity, leading to variable performance across different races, genders, or regions. Previous analyses have shown that a substantial proportion of retinal disease AI studies inadequately report participant demographic characteristics, thereby limiting transparency and generalizability (56). Similarly, glaucoma prediction models that do not explicitly address sensitive attributes may unintentionally amplify disparities in high-risk populations (57). When such biased data are incorporated into educational models, trainees may develop skewed perceptions when encountering diverse patient populations. For instance, an AI diagnostic tool trained predominantly on fundus images from East Asian populations may exhibit significantly lower accuracy in identifying early-stage glaucoma in patients of African descent, who have a higher risk and often present with different optic disc features. A resident trained with such a biased tool in a diverse community clinic may fail to develop the necessary skills to screen high-risk populations, inadvertently leading to missed diagnoses and exacerbating health disparities. Addressing this requires not only building diverse and representative datasets but also mandating that model developers transparently report performance across various demographic subgroups, ensuring trainees are fully aware of the inherent limitations and potential biases of the AI tools they use.

Data privacy and security further complicate the integration of AI into ophthalmic education. Image features extracted by foundational AI models may contain information capable of re-identifying patients, thereby posing privacy risks in educational data sharing and federated learning (58, 59). Striking a balance between leveraging real clinical data for training and rigorously protecting patient privacy remains an essential technical and ethical challenge. Robust de-identification strategies, secure data governance frameworks, and clearly defined consent mechanisms are therefore essential prerequisites for responsible implementation.

Practical barriers related to infrastructure and legal accountability also limit real-world deployment. This highlights a critical divergence in AI adoption pathways between different resource settings. In resource-limited regions, AI has the potential to compensate for expert shortages, yet hardware and network limitations remain bottlenecks (60). The lack of AI educational resources in low-income areas, compounded by inadequate support for teachers and hardware, intensifies existing educational disparities (61). For instance, while high-resource settings can invest in high-fidelity VR surgical simulators with haptic feedback, their implementation is often impractical in low-resource areas due to prohibitive hardware costs and network limitations. Conversely, more accessible solutions, such as AI-powered analysis of surgical videos for MSICS, offer a more feasible and immediately impactful means for standardizing education where expert trainers are scarce. This disparity underscores that a one-size-fits-all approach is insufficient; future efforts must focus on developing context-aware AI tools tailored to local infrastructure to truly bridge the global education gap.

Finally, unresolved questions surrounding legal liability pose a significant barrier to curricular integration. When educational errors arise from flawed AI-generated content, responsibility among model developers, educational institutions, supervising clinicians, and trainees remains poorly defined. This legal ambiguity may deter the formal adoption of AI tools within accredited training programs (3). Establishing clear regulatory guidance and accountability frameworks is therefore essential to support the safe, ethical, and sustainable incorporation of AI into ophthalmic education.

## 8 Limitations

Several limitations should be acknowledged when interpreting this review. First, this study adopts a macro-level perspective, focusing on overarching applications, challenges, and future directions of artificial intelligence in ophthalmic education. As a result, it does not explore detailed algorithmic architectures, model optimization strategies, or dataset-level characteristics, which may limit its direct applicability for technical developers or implementation-focused researchers.

Second, the field of AI-assisted ophthalmic education is still emerging and rapidly evolving. The available literature is therefore limited in volume and heterogeneous in terms of AI models, educational contexts, and outcome measures, precluding direct comparison or quantitative meta-analysis. In addition, publication bias cannot be excluded, as studies reporting positive findings are more likely to be published. The included studies are also highly heterogeneous in terms of AI models, educational settings, and evaluation metrics, which prevents direct comparison or quantitative meta-analysis. The majority of included studies are small-scale, single-center investigations with short follow-up periods, and the long-term effectiveness and generalizability of these educational interventions remain uncertain.

Finally, our literature search was restricted to peer-reviewed, English-language publications, which may have resulted in the exclusion of relevant studies from grey literature or non-English sources, thereby limiting the overall comprehensiveness of this review.

## 9 Conclusion and future perspectives

Artificial intelligence is rapidly reshaping ophthalmic education, spanning large language model-assisted theoretical learning and content generation, computer vision-based objective assessment of surgical skills, AI-supported clinical reasoning training, and patient health education. Collectively, these technologies are fostering a more efficient, standardized, and personalized educational ecosystem, while offering a scalable approach to mitigating global disparities in ophthalmic training resources.

Looking forward, AI in ophthalmic education is expected to evolve toward greater intelligence, multimodality, and embodiment. Multimodal foundation models will increasingly integrate text, imaging, and genomic data to simulate complex cases that closely mimic real clinical scenarios (62–64). A feasible technical path for this involves using cross-modal attention mechanisms to map heterogeneous data types such as text, imaging, and genomic data into a joint embedding space. This would enable the model to generate nuanced, patient-specific scenarios that reflect real-world clinical complexity. Embodied AI holds promise for deeper integration in surgical robotics and virtual reality training, providing haptic feedback and real-time interaction to further narrow the gap between simulation and clinical practice (65). However, the translation of these innovations into routine educational use will depend not only on technical progress but also on responsible governance.

To realize this vision, it is imperative to address the current ethical and implementation challenges. Establishing rigorous AI content review mechanisms, ensuring data diversity and fairness, and developing clear frameworks for privacy protection and liability are prerequisites for safe and responsible integration of AI into ophthalmic education. Ultimately, AI should be viewed as an assistive educational partner rather than a replacement for human expertise. Future ophthalmologists will need to develop not only strong clinical competencies but also the ability to critically evaluate and collaborate with AI systems, ensuring that technological advances are harnessed to enhance training quality and, most importantly, patient care.
